# Assessing Public Policies and Assets That Affect Obesity Risk While Building New Public Health Partnerships, New Hampshire, 2011

**DOI:** 10.5888/pcd10.120349

**Published:** 2013-08-08

**Authors:** Ludmila Anderson, Scot Foster, Regina Flynn, Mindy Fitterman

**Affiliations:** Author Affiliations: Scot Foster, Regina Flynn, Mindy Fitterman, New Hampshire Department of Health and Human Services, Concord, New Hampshire.

## Abstract

The New Hampshire Obesity Prevention Program and the 9 New Hampshire regional planning commissions assessed the state’s obesity-related policies and assets by using community measures recommended by the Centers for Disease Control and Prevention. A self-administered questionnaire that focused on policies and assets that promote healthful eating, physical activity, and breast-feeding was sent to 234 municipalities; 59% responded (representing 73% of the state’s population). Of the municipalities that responded, 52% had sidewalks, 22% had bicycle lanes, none had nutrition standards, and 4% had a policy supporting breastfeeding. Through collaboration, we gathered baseline information that can be used to set priorities and assess progress over time.

## Objective

Policy and physical assets (eg, trails, playgrounds) are important elements in obesity reduction (1,2). To guide public health practice related to obesity reduction, the Centers for Disease Control and Prevention released *Recommended Community Strategies and Measurements to Prevent Obesity in the United States: Implementation and Measurement Guide*, also called the Measures Project (MP) (3). The MP recommends strategies and measures that support and promote healthful eating and physical activity in towns and cities. The objective of our study was to assess the extent to which MP-recommended policies and assets already exist in New Hampshire municipalities.

## Methods

The New Hampshire Obesity Prevention Program (NHOPP), in collaboration with New Hampshire’s 9 regional planning commissions (RPCs), designed a cross-sectional survey using MP questions on policies and assets related to healthful food and beverage choices, physical activity, and breastfeeding. The selected questions were those that the NHOPP considered best suited for a baseline assessment and for a future evaluation of progress. Because the intended respondents were municipal representatives, questions related to schools or school environments were excluded. Any city or town with its own corporate status and local government was considered a municipality.

The survey instrument adhered to the original language of the MP as closely as possible; however, some questions were modified, and a question related to clearing snow from sidewalks was added. Modifications were related to the self-administration, target audience, and specific aspects of our largely rural state. Some questions were simplified, and other questions had example responses. Questions about information that could be gathered from the RPCs were not included in the survey.

To ensure relevance to city and town respondents, representatives of the RPCs previewed and provided input on the survey instrument. RPCs also assisted with distribution and data collection by sending the survey directly to town and city contacts in their respective regions. The survey was disseminated electronically by using the online tool SurveyMonkey (SurveyMonkey, Palo Alto, California). Data were collected from March through December 2011; nonrespondents received 2 electronic reminders and a telephone call. Municipal representatives received a printed copy of the survey (Appendix) and had the option to fax or mail their responses to the NHOPP. Each RPC provided data on the total population and acres of land in each municipality in its region. Data were analyzed using SAS software version 9.2 (SAS Institute Inc, Cary, North Carolina). Proportions of municipalities reporting assets and policies were calculated with “I do not know” responses included in the denominators.

## Results

Of 234 municipalities, 137 (59%) responded to the survey, representing 53% of the state’s surface area and 73% of the state’s population. Respondents were mainly town administrators and administrative assistants. Of the 137 respondents, 112 (82%) finished the survey; 104 (76%) answered every question. Of municipalities that responded to the relevant questions (Table 1), 52% reported having sidewalks, 39% areas for mixed-use development, 21% designated shared-use paths and bike lanes, 37% medium-to-large grocery stores, and 34% policies to encourage local agriculture. No municipality reported having nutrition standards or policies related to selling or advertising unhealthful foods or to encouraging healthful eating.

**Table 1:** Assets or Policies Related to Physical Activity and Nutrition in Municipalities, New Hampshire, 2011

Asset or Policy	No. of Respondents	No. (%) of Respondents With the Asset or Policy
Have designated shared-use paths and bicycle lanes	121	26 (21)
Have areas zoned for mixed-use development	112	44 (39)
Have sidewalks	123	64 (52)
Require prompt clearing of snow from sidewalks	64	39 (61)
Require building sidewalks and bicycle lanes with new construction	119	20 (17)
Have partnerships that address healthful eating and active living	113	21 (19)
Have nutrition standards	117	0
Prohibit advertisement of less healthful choices	115	0
Prohibit sale of less healthful choices	116	0
Limit or reduce portion sizes	116	0
Reduce cost of more healthful foods and beverages	116	0
Have medium to large grocery stores	109	40 (37)
Have community gardens	113	33 (29)
Have farmers’ markets	104	57 (55)
Encourage local agriculture	113	38 (34)
Provide time and space for breastfeeding employees	114	5 (4)

Altogether, 97 municipalities reported having 5,724 miles of paved streets; 20 of those municipalities reported having 211 miles (4% of the reported miles of paved roads) of shared-use paths and bike lanes. A total of 47 municipalities reported having 1,347 miles of sidewalks (24% of reported miles of paved streets), and 23 reported having 66,338 acres of land zoned for mixed-use development (2.5% of total zoned acres in all reporting municipalities). In addition, 33 municipalities reported having 83 community gardens (1.5 per 10,000 residents of those 33 municipalities), 57 municipalities reported having 100 farmers’ markets (1.6 per 10,000 residents of those 57 municipalities), and 40 reported having 108 medium-to-large grocery stores (1.7 per 10,000 residents of those 40 municipalities). Twenty-one municipalities reported having partnerships that address physical activity or healthful eating. Some of those partners are local government centers, local businesses, and parks and recreation programs. To questions about outdoor recreational facilities, most responding municipalities reported having walking and hiking trails (86%), snowshoe and cross-country ski trails (70%), open-play fields (87%), and community playgrounds and parks (84%) (Table 2).

**Table 2:** Reported Recreational Facilities in Municipalities, New Hampshire, 2011

Facilities	No. of Respondents	No. of Facilities		
		1–10, n (%)	>10, n (%)	None, n (%)
Walking and hiking trails	111	73 (66)	22 (20)	16 (14)
Snowshoe and cross-country ski trails^a^	106	57 (54)	17 (16)	27 (26)
Bicycle trails^a^	103	44 (43)	4 (4)	50 (49)
Open play fields^a^	113	89 (79)	9 (8)	14 (12)
Public pools	106	24 (23)	0	82 (77)
Community playgrounds and parks	112	87 (78)	7 (6)	18 (16)

a Responses of “I do not know” are not shown.

## Discussion

Although only 59% of municipalities responded, most (73%) of the New Hampshire population resides within those municipalities. Survey findings allow communities to set priorities, plan improvements, and reassess their policies and assets in 5 years.

Numerous initiatives are under way: one example is the launch of the New Hampshire Livable Walkable Communities Toolkit (4), a comprehensive needs assessment to set goals and priorities for regional planning and the imminent implementation of identified strategies, and projects done by community partners. Most municipalities have walking, hiking, and other built-environment assets that support and encourage daily physical activity. Although no municipality reported nutrition standards or policies related to healthful eating, 4% of responding municipalities have a policy supporting employees who breastfeed.

Limitations of our assessment stem from the survey being self-administered, which influenced survey length and content. Although no question was consistently unanswered, open-ended questions were most often skipped. The response rate and appreciation of the survey purpose could have been improved by advance promotion of the survey among municipal representatives. However, the involvement of regional planners improved survey accuracy and shortened the questionnaire.

Survey distribution by the RPCs added credibility to the assessment. In addition to acquiring baseline data, the NHOPP built a partnership with the RPCs, which is invaluable for planning and implementing our shared goal to create environments that support healthful eating and active living.

## Appendix. Survey of Municipal Policies and Assets Related to Physical Activity and Nutrition

1. What municipality do you represent?

2. What is your job title?

3. Approximately, what is the total mileage of paved streets managed and paid for by your municipality (excluding limited access highways)?

4. Does your municipality have any designated shared-use paths and bike lanes?

5. If you answered “yes” to the previous question, approximately, what is the total mileage of shared-use paths and bike lanes within your municipality?

6. Does your municipality have any sidewalks?

7. If you answered “yes” to the previous question, approximately, what is the total mileage of sidewalks within your municipality?

8. Does your municipality have a policy regarding the prompt clearing of snow from sidewalks?

9. Does your municipality have any policy related to new road construction that would require building of sidewalks or bike lanes?

10. If yes, please describe:

11. Does your municipality have any areas zoned for mixed-use development?

12. If you answered "yes" to the previous question, approximately, what is the total number of acres zoned for mixed-use development?

13. Does your municipality have any of the following outdoor recreational facilities?

- Walking/hiking trails
- Snowshoe/cross-country ski trails
- Bicycle trails
- Open play fields
- Public pools
- Community playgrounds or parks

14. If your municipality has additional (indoor or outdoor) recreation facilities that were not counted in the previous question, please list all that apply.

15. Does your municipality have any policies or zoning regulations that encourage local agriculture?

16. If yes, please describe:

17. Does your municipality have any community gardens?

18. If yes, approximately, how many community gardens are located in your municipality?

19. Approximately, how many farmers’ markets operate within your municipality in a given year? (Each location = one market)

20. Approximately, how many medium to large grocery stores are located within your municipality?

21. Not counting schools, does your municipality have any policy that prohibits *advertising* and promotion of less healthy foods and beverages in municipal buildings?

22. If yes, please describe:

23. Not counting schools, does your municipality have any policy that prohibits the *sale* of less healthy foods and beverages in municipal buildings?

24. If yes, please describe:

25. Not counting schools, does your municipality have any policy to limit the *portion size of entrées* sold in municipal buildings (including sandwiches and salads) for example by reducing the standard portion size of one or more entrées?

26. If yes, please describe:

27. Not counting schools, does your municipality have nutrition standards that are consistent with the *Dietary Guidelines for Americans* for food sold in municipal buildings? This includes foods sold in cafeterias and/or vending machines.

28. If yes, please describe:

29. Not counting schools, does your municipality have any policy to reduce the cost of healthier foods and beverages sold in municipal buildings such as selling water at a cheaper price than sugar-sweetened beverages?

30. If yes, please describe:

31. Does your municipality have any policy that requires provision of both, time and a designated private space (not a bathroom stall), in municipal buildings to allow breastfeeding employees to pump breast milk during work hours?

32. If yes, please describe:

33. Does your municipality have any coalitions or partnerships that address active living and/or healthy eating?

34. If yes, what are the names of the coalitions?

35. Is there anything you would like to add?

Thank you!
